# The Record for 1886

**Published:** 1885-11-20

**Authors:** 


### THE RECORD FOR 1886.

Our journal for 1886 will in no respect fall behind its former excellence, but will be more than ever adapted to the wants of the busy Practitioner. Our aim has been to furnish the profession with a plain, though neat, *practical* journal ; not so cheap as to be trashy, nor so high-priced as to be beyond the means of those members of the profession who are compelled to use economy. Our journal aims at the medium or middle ground, that will adapt it to the wants of the great majority of Medical Practitioners. One of those (Dr. Akers, of DeKalb County, Ga.) seems fully to understand and appreciate our design in the following extract from a letter addressed to the Managing Editor : "I like the size and plan of your journal. It is just the thing for the busy practitioner. The terms are certainly very cheap for the evident care and judgment bestowed upon its contents, in which is a brief of the entire field of medical literature."

Dr. Pope, of Clarke County, Ga., an old subscriber, in a recent letter, says : "No journal compares with the SOUTHERN MEDICAL RECORD *for the busy practitioner*. I like it better each succeeding year. It deserves unbounded success."

Hundreds of testimonials equally complimentary to the RECORD could be given ; but we feel this to be unnecessary. Read next article.

